# Role of human antigen-specific T cells in tolerance and IgE-mediated allergic reactions to food

**DOI:** 10.3389/fimmu.2025.1595696

**Published:** 2025-06-12

**Authors:** Rosina López-Fandiño

**Affiliations:** Instituto de Investigación en Ciencias de la Alimentación (CIAL, CSIC-UAM), Madrid, Spain

**Keywords:** food allergy, allergen-specific cells, pathogenic effector T cells, specific regulatory T cells, specific immunotherapy

## Abstract

Allergen-specific CD4^+^ T cells play a pivotal role in the pathophysiology of food allergy and in the induction of tolerance during desensitization. However, their study remains challenging due to the considerable heterogeneity in the frequency and phenotype of food allergy-specific Th2 cells in allergic individuals, together with an extremely low abundance of T cells specific to a given allergen. Furthermore, a major limitation of human studies is their reliance on peripheral blood sampling, whereas higher frequencies of allergen-specific cells may reside in intestinal tissues directly implicated in the allergic response. Among the most targeted approaches to quantify and characterize these cells stand MHC class II tetramers and activation-based detection methods. These techniques have identified pathogenic Th2 cell subpopulations in allergic individuals, that are absent in non-allergic subjects and differentiate patients with diverse degrees of clinical reactivity. These cells, crucial in driving immune responses and mediating immunological memory, exhibit distinct phenotypic and functional properties compared to conventional Th2 cells. However, there is no conclusive evidence supporting a definitive role for allergen-specific regulatory T (Treg) cells in food allergy, natural tolerance, or desensitization following immunotherapy. Moreover, pathogenic Th2 and Treg cells may differ in their allergen specificity, potentially due to priming by distinct sets of food-derived antigens. A deeper understanding of the characteristics and roles of specific pathogenic Th2 and Treg cell populations in food allergy could pave the way for novel preventive and therapeutic strategies for this disease.

## 1 Introduction

Allergen-specific CD4^+^ T cells play a major role in the physiopathology of food allergy as well as in tolerance induction during desensitization (1). However, their study is complex due to the great heterogenicity of the Th2 cell response in allergic subjects (2), as well as the existence of an extremely low frequency of T cells specific for a particular allergen (approximately 10^-4^−10^-7^ in the naïve repertoire and 5x10^-2^−10^-5^ in the memory repertoire; 3). Antigen-specific cells have been characterized by screening the proliferative responses, intracellular cytokine expression, and cytokines released by stimulated PBMCs from allergic patients, using flow cytometry, ELISA or ELISPOT (4–9). Nevertheless, proliferation assays often select the more expansive clones, change the cellular phenotype and function, and cause bystander multiplication (10). On its part, as with intracellular cytokine staining, cytokine detection by ELISPOT allows visualization of the secretory products of individual activated cells, and thus, the identification of the number of functional T cells and their effector function. However, the assay provides limited information, requires long incubation times and it is difficult to detect several cytokines at a time (11). These shortcomings highlight the need for more sophisticated targeted approaches to identify, characterize and enumerate specific T cells (12), especially considering that the frequency of food allergen-specific cells is lower than that of cells reactive to antigens associated to chronic or repeated exposure, probably due to antigen avoidance in food allergic patients (13, 14). This review addresses the main strategies to estimate the frequency and phenotype of antigen-specific cells relevant to food allergy and analyses the contribution of these approaches to the understanding of the mechanisms underlying the establishment of food allergy and its resolution.

## 2 Methods to monitor antigen-specific cells in human subjects

The MHC multimer technology recognizes antigen-specific CD4^+^T cells at a single cell level and allows the monitoring of the surface phenotype of reactive cells, although it has the disadvantage of being restricted to a small number of epitopes in major allergens (it does not detect cells directed to peptides not included in the assay) and to particular HLA genotypes (that express MHC class II molecules able to recognize and bind those particular epitopes). Due to the high polymorphism of HLA genes, identifying suitable peptide epitopes for specific MHC class II molecules is inherently challenging, and the technical complexity of producing peptide-loaded MHC class II multimers further complicates the analyses. MHC class II tetramers complexed to peanut epitopes were used to examine the frequency and phenotype of specific CD4^+^ T cells in peanut allergic individuals (14) and in peanut allergic patients with different degrees of clinical reactivity (15). It should be noted that the expression of certain surface activation markers or receptors is influenced by both antigen presentation and T cell stimulation. Therefore, while tetramer staining is a valuable tool for identifying antigen-specific T cells, it may not fully capture the functional diversity or activation status of these cells (16), or exhibit a strict correlation with assays measuring activation marker expression (17). However, studies suggest that when identical epitopes are used, tetramer staining and the CD154 assay yield comparable frequencies and phenotypes (18).

The identification of allergen-responsive T cells using activation-based detection approaches relies on *in vitro* stimulation with a crude allergen extract containing all relevant allergens and it is independent of the individual HLA status. Furthermore, this method allows for a sufficiently short stimulation period, enabling efficient antigen processing by antigen-presenting cells (APCs) and subsequent T cell activation while preventing the expansion of allergen-responsive T cells. Activation markers are then stained with fluorophore-conjugated antibodies and detected by flow cytometry (19). This procedure can be followed by magnetic or fluorescence-activated cell sorting and coupled with single-cell RNA-seq and paired T cell receptor (TCR) sequencing to dive into the heterogenicity of patients with food allergy (3, 20, 21).

Expression of CD154 (also named CD40L), a member of the tumor necrosis factor (TNF)-ligand superfamily, by activated T cells is highly sensitive and selective for TCR engagement (22). The CD154 assay serves as a valuable tool not only for assessing specific T cell responses to food allergens, but also for characterizing CD4^+^ T cells reactive to bacterial antigens. This dual functionality provides a promising avenue for investigating intestinal T cell responses to the microbiota, with broad implications for understanding mechanisms underlying food allergy (23). Assessment of antigen-specific T cells according to CD154 expression can be performed extracellularly with a blocking antibody against CD40 (24). This approach prevents the interaction between CD154 and CD40 on APCs, which would otherwise lead to downregulation and degradation of CD154. By stabilizing CD154 surface expression, this method enables subsequent staining with a fluorescently labeled anti-CD154 antibody for analysis. Alternatively, the addition of a fluorescent antibody against CD154 during stimulation also allows its detection on the cell surface, being equally compatible with further cell characterization or sorting, although CD154-antibody complexes may become unstable, potentially leading to an underestimation of the proportion of responding cells (25). CD154 can also be detected intracellularly with the addition of Brefeldin A, which blocks the transport of proteins to the cell surface and allows the simultaneous analysis of cytokines, providing higher frequencies of CD4^+^CD154^+^ cells than extracellular detection methods, although it is not compatible with further multiparameter analyses (22, 24).

The extremely low frequencies in which T cells specific for a particular antigen are present in the periphery underscore the need for appropriate surface markers additional to CD154 for their precise identification. Various transmembrane proteins and costimulatory molecules, including OX40, CD69, and CD25, are upregulated on the cell surface following CD4^+^ T cell activation via TCR and CD28 signaling by APCs (26, 27). However, CD154 exhibits greater specificity than CD69 and it is upregulated more rapidly than OX40 and CD25, thereby minimizing bystander activation (22). Of note, expression of CD69 and CD25 allowed the detection, in peanut allergic patients, of allergen-specific CD8^+^ T cells (28). Among these markers, CD137, a member of TNF-receptor superfamily (also named TNFRSF9 or 4-1BB), allows a distinction between regulatory T (Treg) cells and effector T cells and thus their simultaneous analysis (29, 30). Thus, CD137^+^CD154^-^ expression enabled the identification and sorting, after prior expansion, of a Treg population with high suppressive potential and epigenetic stability (31). Furthermore, assays that integrate and simultaneously analyze 4 activation-induced markers: CD154, CD137, CD69 and OX40 could overcome the possible biases related to the selection of a single pair of markers, enhancing the sensitivity of determination of CD4^+^ T responses while maintaining high specificity (32).

## 3 Allergen-specific pathogenic CD4^+^T cells

Even though, as mentioned, Th2 cells linked to food allergy are highly heterogeneous, several researchers, using tetramer staining or activation markers, have highlighted the existence in allergic subjects of pathogenic Th2 cell subpopulations which share analogous features yet display distinct phenotypic and functional characteristics compared to conventional Th2 cells (**Table 1**). Thus, peanut and egg allergy have been reported to be characterized by the presence of allergen-specific, terminally differentiated memory (CD45RA^-^CD45RO^+^CD27^-^) CD4^+^ T cells with a Th2 phenotype, referred to as Th2A (34), T follicular helper 13 (Tfh13; 39), Th2^+^ (36), or, as a whole, type 2 effector cells (38). Allergen-specific Th2 cells were also identified in patients allergic to other foods, such as shrimp, walnut, cashew or cow’s milk (33, 42–44).

**Table 1:** Relevant characteristics of allergen-specific CD4^+^ T cells related to food-mediated allergic disease present in human PBMCs.

Subtype	Methodology of detection	Phenotypic profile	Functional attributes	Relevance to allergic disease	Reference
Walnut-specific T cells	Peptide (Jug r 2) MHCII tetramer staining and generation of T cell clones	-CD27^+^CCR4^+^CRTH2^-^ CCR7^+^CD62^+^ -CD27^-^CCR4^+^CRTH2^+^	-Central memory Th2 cells -Effector Th2 cells with 3 different profiles: Th2 (IL-4, IL-5, IL-13, *GATA3*), Th2/Th17 (IL-4, IL-17A, *GATA3, RORC*), and Th17 (IL-17, *RORC*)	High frequencies in walnut allergy Absent (or not detected) in non-allergic subjects	Archila et al. (33)
Th2A	Peptide (Ara h 1 and Ara h 2) MHCII tetramer staining	CD45RO^+^CCR4^+^CD200R^+^ CD58^+^CD29^+^CRTH2^+^ CD161^+^CD49d^+^CD27^low^ CD45RB^low^CCR7^low^CD7^low^	Stable, simultaneous production of multiple Th2 cytokines High expression of *IL5, IL9, IL17RB, ST2, CLRF2, hPGDS, PTGS2, GPR42*, and *PPARG)*	High frequencies in peanut allergy (also in perennial, mould and pollen allergy) Absent in non-allergic subjects Th2A cells are specifically activated after peanut OFC	Wambre et al. (34)
Th2A	Surface expression of CD154 after 18 h of stimulation with peanut extract	CD154^+^CD45RA^-^CD27^-^CRTH2^+^CD161^+^ST2^+^ IL17RB^+^	Antigen-specific cell activation causes transient surface expression of epithelial cytokine receptors and leads to increased IL-4 and IL-5 production	Absent in non-allergic subjects	Calise et al. (16)
Peanut-specific T cells	Surface expression of CD154 after 14 h of stimulation with overlapping Ara h 1, 2, 3, 6, 8 peptides	-CD154^+^CRTH2^+^CD161^+^ CD27^-^ -CD154^+^CCR7^+^CRTH2^lo^ CCR6^hi^ CD27^hi^ -CD154^-^CD25^+^FOXP3^+^ CD137^+^CD127^-^OX40^+^	-Th2A. High expression of *IL4, IL13*, *IL5, IL9, IL17RB, IL1RL1, GATA3*, and *PPARG* -Central memory differentiation state. High expression of *IFNG, RORC*, *IL17A, IL17F, IL23R1, FOXP3* and *L22* (Th1/Th17/Treg-related genes) -Treg cells	-Positive correlation with specific IgE and response to a DBPCFC with 100 mg of peanut protein -Negative correlation with specific IgE and response to a DBPCFC with 100 mg of peanut protein -No differences between responders and non-responders	Bajzik et al. (35)
Th2^+^	Surface expression of CD154 after 6 h or 18 h of stimulation with peanut extract	CD154^+^IL-4^+^IL-13^+^CCR4^+^ CCR6^+^CXCR5^+^hPGDs^+^ CD45RO^+^CD45RA^-^ CD27^low^CCR7^low^ (6 h)	High expression of *IL4, IL5, IL9, IL13*, *IL3, CSF2*, and *IL17RB* (18 h)	Specific for clinical reactivity to peanut Significant correlation with peanut-specific IgE	Chiang et al. (36)
Type 2 cells	Surface expression of CD154 after 6 h of stimulation with egg white protein	CD154^+^IL-4^+^IL-13^+^CCR4^+^ CXCR5^+^	Type 2 cytokine expression negatively correlates with CCR6 expression	Significant correlation with egg-specific IgE Type 2 cell responses are associated with baked egg tolerance	Berin et al. (37, 38)
Tfh13 cells	Surface expression of CD154 after 6 h of stimulation with peanut extract	CD154^+^CXCR5^+^GATA3^+^ IL-4^+^IL-13^+^	Transcriptionally distinct from Tfh2 and Th2 cells	Absent in non-allergic subjects Responsible for the generation of high affinity, anaphylaxis-inducing IgE	Gowthamanan et al. (39)
Peanut-specific T cells	Surface expression of CD154 and CD137 after 20 h of stimulation with peanut extract	-Strong upregulation of modules representing Th2, Th1 and Th17 signatures in both CD154^+^ CD137^-^ and CD154^-^ CD137^+^ compartments -CD137^+^ compartment	-Tfh2-like (*CXCR5, PDC1, IL5, Il4, ITGA4* and *PLA2G16)*) Th2A-like (*GATA3, IL17RB, PTGDR2*) Th2 regulatory-like (*FOXP3, TNFRSF9*) Tfh1-like Th1conv Th17 -Tfh-like Tregs (*IL10*) Conventional Tregs CCR7^+^ Tregs	-Tfh2-like correlate with peanut specific IgE Th2A-like do not correlate with specific IgE Th2 regulatory-like are deviated Treg cells	Monian et al. (20)
Peanut-specific T cells	Surface expression of different activation markers (CD154, CD137, OX40, CD69 and CD25), after 6, 24 or 48 h of stimulation with peanut extract	-CD154^+^CD69^+^FOXP3^-^ CD27^-^CCR7^-^CCR6^-^ CXCR3^-^CCR4^+^CRTH2^hi^ PDI^hi^ (6-48h) -CD137^+^CD154^-^FOXP3^-^ CD27^+^CCR7^-^CCR6^-^ CXCR3^+^CCR4^-^PD1^hi^HLA-DR^hi^ (24-48h) -CD137^+^CD154^-^FOXP3^+^ CD27^+^CCR7^+^CCR6^+^ CXCR3^-^CCR4^+^PD1^hi^HLA-DR^hi^ (24-48h) -CD25^+^OX40^+^FOXP3^+^ CD27^+^CCR6^+^ CXCR3^-^CCR4^+^CD62^+^ IRF4^low^PD1^low^ (24-48h)	-Terminally differentiated mature effector memory Th2 cells (high secretion of IL-4, IL-5, IL-13 and IL-2) -Effector memory Th1 subset (secretion of IFN-γ, TNF-α, and IL-2) -Memory Treg-associated subset (secretion of IL-10). Suppressive on Th2 cells *in vitro* -Memory Treg phenotype (no specific cytokine secretion). Suppressive on Th2 cells *in vitro*	Early secretion of IL-2 by peanut-specific Th2 cells promotes efficient and durable activation of Treg cells after *in vivo* recognition in allergic subjects	Lozano-Ojalvo et al. (27)
Peanut-specific Tregs	Surface expression of CD154 and CD137 after 7 h of stimulation with peanut extract	CD154^-^CD137^+^FOXP3^+^	Suppressive properties and stable demethylation status of the *FOXP3* locus No production of effector cytokines	Frequency and stability independent from the allergic status	Weissler et al. (40)
Ovalbumin-specific Tregs	Surface expression of CD137 after 6 h of stimulation with ovalbumin	CD137^+^IL-10^+^ and CD137^+^FOXP3^+^	Activated memory cells	Age-dependent expansion in infants subjected to early egg introduction and in those not allergic to egg at the age of 12 months CD137^+^IL-10^+^ cells correlate with specific IgG4 at 12 months	Lai et al. (41)
Cow’s milk specific T cells	Surface expression of CD154 and CD137 after 6 h of stimulation with a peptide pool	CD45RA^-^FOXP3^+^ CD25^+^CCR7^-^CD127^-^	Differentially activated subset of allergy-associated effector T cells with no regulatory function. High expression of *FOXP3, CD25, TIGIT, IL32, CD247, S100A6, S1000A10, TNFRSF1B, CXCR4*	Increased in baked-milk allergy with respect to healthy controls, with baked-tolerant patients showing an intermediary phenotype	Lewis et al. (42)

OFC, oral food challenge, DBPCFC, double blind placebo-controlled food challenge.

These cells distinctively express a large amount of a broad spectrum of Th2 cytokines, the receptors for the epithelial alarmins IL-33 and IL-25 (ST2 and IL-17RB), which indicate that they are activated at inflamed mucosal sites, and hematopoietic prostaglandin D synthase (hPDGS) (16, 34, 36). They are also enriched in surface markers for homing to B cell follicles (CXCR5), the small intestine (CCR6), skin and lungs (CCR4) (36), to drive IgE class-switching (20) or expand in the affected tissues, contributing to allergic inflammation at the site of disease (45). While the skin homing markers CLA and CCR10 are not different on peanut reactive T cells with respect to total CD4^+^ T cells, the observation that the chemokine receptor CCR4 is enhanced suggests priming after exposure through the skin and the airways (14, 40, 46). Surprisingly, expression of the gut homing integrin β7 does not distinguish between peanut allergic and non-allergic subjects (14, 46), while CCR6 is negatively correlated with pathogenic type 2 responses (35, 38). These observations suggest either that Peyer’s patches and mesenteric lymph nodes are not the primary sites of initial antigen encounter or that cells primed at intestinal lymphoid tissues do not subsequently recirculate in peripheral blood. Expression of other markers, such as CD161, which identifies Th17 and NK cells involved in inflammatory diseases (47), and the prostaglandin D2 (PGD2) receptor CRTH2 (chemoattractant-homologous receptor expressed on Th2 cells), varies depending on the detection approach (16, 36) and, notably, on the kinetics of CD4^+^ T cell activation (27). CRTH2 expression is associated with repetitive antigen stimulation, and thus, with low expression of CD27 and a terminally differentiated phenotype (33, 43).

Allergen-responsive cells play a central role in facilitating immune responses and in mediating immunological memory. Thus, there is a strong relationship between the pathogenicity of these cells and their maturation stage (34). Recently, Kratchmarov et al. (48) identified a Th2 cell multipotent progenitor in several tissues with the potential to maintain and replenish effector Th2 cell populations, which could contribute to the renewal of these specific, mature cells, with limited proliferative capacity. Upon subsequent encounter with their specific allergen, TCR recognition causes upregulation of surface receptors for epithelial alarmins that considerably enhances the secretion of Th2 cytokines (16). Specific Tfh13 cells, CXCR5^+^BCL6^+^GATA3^+^ cells producing, not only IL-4, but also IL-13, are involved in guiding B cell isotype switching to high affinity IgE in allergy to peanut (39) and milk (49). Notably, in eosinophilic esophagitis (EoE), a type 2 inflammation-driven disorder triggered by food proteins, milk-responsive Tfh cells, which produce IL-10 and low levels of IL-4, support class switch to IgG4 and contribute to distinct clinical manifestations (49). However, while associational observations point at a role of these cells in the maintenance of IgE levels following sensitization (18, 36–38), the abundant expression of the Th2-related genes *IL5, IL13, IL9* and *hPGDS* indicates their implication in additional mechanisms of clinical reactivity beyond IgE-class switching (34, 36, 45, 50). On its part, hPGDS coordinates the expression of cyclooxygenase-2 and the synthesis of PGD2 in human Th2 cells. PGD2 exerts inflammatory effects through high affinity interactions with the G protein coupled receptors DP1 and CRTH2 (also known as DP2), that together promote a number of biological effects associated with the development and maintenance of the allergic response (51). CRTH2 expressed by Th2 cells mediates the activation and recruitment of these cells during the late phase of the allergic response, contributing to their effector function. On its part, hPGDS identifies a minor Th2 subpopulation with proeosinophilic properties and high correlation with multiple allergic inflammatory disorders, such as asthma, atopic dermatitis or eosinophilic gastrointestinal disease (52).

The influence of Th17-related factors (CCR6, CD161, or IL-17 genes) is controversial (33, 34, 45). The finding that the expression of the Th2A-related marker CRTH2 and the Th17-related marker CCR6 is mutually exclusive, and that some patients manifest clinical allergy symptoms in the absence of peanut-specific CRTH2^+^CD27^-^ T cells with a dominance of CCR6^+^CD27^+^ T cells, prompted the hypothesis of the existence of 2 different cellular and molecular signatures, or immunotypes, with different effector and homing potential, associated with food allergy (53). Th17 cells potentiate allergic inflammation through multiple mechanisms (54), although it has also been suggested that, in view of the plasticity of these cells, Th17-like cells could be a precursor or immature form of allergen-specific Th2 cells (38). In fact, the predisposition of Th17 cells to upregulate IL-4 and become Th2 cells has been associated with the exacerbation of allergic diseases (55).

Allergen-specific pathogenic Th2 cells are absent in non-allergic subjects and differentiate patients with diverse degrees of clinical reactivity to peanut (45) or tolerance to backed egg (38). However, a substantial heterogeneity has been reported in the frequency and phenotype of peanut or egg-specific T cell responses in allergic patients, linked to the clinical manifestation of the disease or the nature of the allergen (18), which restricts their value as clinical biomarkers (38). In this respect, it should be noted that these studies in humans have focused on characterizing these cells in peripheral blood, despite that higher frequencies of pathogenic cells may exist in the tissues more directly affected by the allergic response (52). Furthermore, specific T cell numbers in blood may depend on whether the allergic patients are strictly avoiding allergen exposure (2).

## 4 About allergen-specific Treg cells

Several reports have shown that a high Treg cell frequency is associated with the maintenance of oral tolerance (56–58), the natural resolution of food allergy (59), or a phenotype of milder clinical allergic disease and favorable prognosis (60, 61). Oral immunotherapy (OIT) also increases the frequency of Treg cells, although only in individuals who succeed to achieve long lasting clinical tolerance (27). However, unlike the case of aeroallergens, where the contribution of allergen-specific Treg cells to tolerance, at least partially, has been demonstrated (10, 62), there is no conclusive evidence for the role of allergen-specific Treg cells in food allergy (63) (**Table 1**). Early introduction of egg was found to increase the proportion of ovalbumin-specific Treg cells, whereas development of egg allergy at 12 months was associated with impaired expansion of these cells (41). Similarly, it was claimed that there is a functional defect in patients with peanut allergy that prevents the induction of peanut-specific IL-10-producing regulatory T type 1 (Tr1) cells (64). Nevertheless, no other studies found evidence of a deficiency of allergen-specific Treg cells or a disrupted specific Treg cell response in peanut allergic patients compared to healthy controls, nor in individuals with different degrees of clinical reactivity to peanut or egg (36, 37, 40, 45).

These discrepancies can be explained by methodological differences between studies due to the use of different T cell activation markers and stimulation times with the allergen to detect responsive cells. Berin et al. (37) and Chiang et al. (36) observed expression of CD154 on CD4^+^ cells bearing regulatory markers (CD25 and FOXP3) after 18 h of stimulation of PBMCs from allergic patients that was absent in control subjects. Upregulation of CD154 by these cells was a delayed, secondary, response to IL-2 released by effector cells activated at 6 h, suggesting that these cells included non-specifically activated Treg cells. Divergences may also arise if patients are subjected to continuous exposure to the allergen, as this may increase the traffic of cells between the draining lymph nodes and the gastrointestinal tract, increasing Treg cell frequency in peripheral blood (65). Alternatively, the finding of increased numbers of Treg cells in allergic patients could result from a feedback loop designed to limit Th2 immune responses, regardless of whether this mechanism is sufficient to control allergic inflammation (66). It should be noted that identification of specific Treg cells has been performed solely on the basis of expression of regulatory markers and lack of cytokine production upon stimulation with the allergen, as their low frequency in peripheral blood makes it difficult to carry out more conclusive suppression assays without previous *ex vivo* expansion (36).

It has been described that Treg cells acquire characteristics of effector Th2 cells and probably display impaired suppressive functions, and even pathogenic functions, in the setting of a strong tissue inflammatory response, such as that typical of EoE (67) and peanut allergy (20). This Th2-cell-like reprograming of Treg cells, that in allergy-susceptible mice is driven by IL-4 production by ILC2s and activated mast cells (56, 68), supports the interpretation that there is a generally dysfunctional Treg cell response in allergic patients. Alternatively, the observation that, in allergy, antigen-specific Th2 responses develop despite the presence of a quantitatively and qualitatively normal Treg cell compartment has been attributed, in the case of aeroantigens, to Th2 cells and Treg cells targeting different proteins (30). Tissue-resident Th2 and Treg cells in active EoE have also been found to have different allergen-specific properties, suggesting stimulation by different sets of food-derived antigens (67). Similarly, in peanut allergy, CD154^+^ and CD137^+^ cells were found to have different TCR repertories, indicating that factors such as TCR affinity or the tissue environment during priming may influence the induction of specific T cell phenotypes (20). These observations may explain why pathogenic Th2 cells seem to exhibit higher resistance to the suppressive effect of Treg cells (36, 37, 40, 45), implying that proteins that stimulate strong Th2 responses fail to activate Treg cell responses, and suggesting that the induction allergen-specific responses would efficiently suppress Th2 development.

In fact, whether allergen specificity is required for Treg functionality is a debated issue. Treg cell maintenance and development depends on IL-2 produced by other T cells and it was shown that Treg cells with diverse TCRβ repertoires activate as result of a bystander-amplified response initiated by IL-2 produced by highly specific (with restricted TCRβ clonal diversity) Th2 effector cells upon allergen recognition (27). This was demonstrated in *in vitro* experiments and also by the persistent activation of Treg cells and expression of immunoregulatory molecules following oral food challenge in peanut allergic subjects (27). Notably, a population of terminally differentiated Treg cells that plays a crucial role in maintaining intestinal health has recently been identified that becomes independent of TCR signaling and acquires an “innate-like” function (69). Once the suppressive activity of Treg cells is triggered, they can inhibit cells of the innate immune system, such as mast cells, basophils and eosinophils (70), as well as other T cells with different antigen specificity, leading to an immunosuppressive albeit non-specific function that has been exploited in Treg cell-based therapies (71). In mice, it was shown that it is possible to induce tolerance to a bystander protein through the establishment of a stable suppressive environment after treatment with another protein (72). This effect, associated with increased Foxp3 expression and IL-10 production, has been hypothesized to occur, at least partially, through the downregulation by Treg cells of APCs that present different antigens to naive CD4^+^ T cells with diverse specificities, hampering their activation (73).

As mentioned, these observations in PBMCs do not exclude the possibility of a local Treg cell accumulation in the gastrointestinal mucosa, only accessible for study when human tissue samples are available through biopsies. Studies in mice, that enable the detailed investigation of antigen-specific cells in contexts that are challenging to study in humans, have identified Treg cells specific for ingested peptides within secondary lymphoid organs associated with the gut-liver axis, with a particularly high prevalence in the lamina propria of the small intestine and colon (74). Following prolonged dietary exposure, α-zein-responsive Treg cells, which arise concomitantly with solid food intake, were found to exhibit a localized distribution in the small intestinal lamina propria of mice (75). This study further demonstrated that immune recognition in the context of food tolerance is dominated by a limited subset of epitopes, which do not appear to correspond to known allergen epitopes (75). In humans, approximately 9% of memory CD4^+^ T cells express FOXP3 in peripheral blood and colonic mucosa, whereas only 2% of lamina propria CD4^+^ T cells in the small intestine of healthy individuals are FOXP3^+^. However, this population increases tenfold under inflammatory conditions, such as active celiac disease (76). Similarly, Wen et al. (67) found an overabundance of effector Th2 cells and Treg-like cells, along with elevated Th2 cytokine expression, in endoscopic biopsies compared to autologous peripheral blood samples, in patients with EoE. Moreover, Treg cells specific for food-derived or commensal bacterial antigens are rare in peripheral blood, despite the presence of high frequencies of peripheral Treg cells responsive to environmental antigens such as plant pollen or house dust mite, which suggests that Treg cells relevant to food allergy predominantly localize within intestinal tissues (30). Tonsils, that are exposed to food allergens before they are digested, contain 3 times more FOXP3^+^ Treg cells than peripheral blood, indicating a potential role as a first line of tolerance induction (77). Palomares et al. (77) further demonstrated an enrichment of Bet v 1-specific Treg cells in tonsils, where they constitute 30% of Bet v1-specific CD4^+^ T cells. However, whether this effect is antigen-dependent (restricted to aeroallergens) or rather reflects the anatomical location or the functional features of this lymphoid organ remains to be elucidated. In fact, aeroallergen and food allergen-sensitized subjects differ in tonsillar gene expression, with the former exhibiting more pronounced immune responses (78).

## 5 Allergen-specific cells in the resolution of food allergy through specific immunotherapy

High frequencies of food-specific pathogenic Th2 cells correlate with adverse clinical parameters and with treatment failure following egg OIT (38, 45) and, according to some studies, successful OIT to peanut parallels a marked reduction in these cells (34), although not all subsets of allergen-reactive cells are equally altered by the treatment (20, 35, 53) (**Table 2**). However, despite that a favorable clinical outcome has been reported to be accompanied by the induction of allergen-specific Treg cells with enhanced function, as determined by proliferation assays (7), other studies using more targeted approaches failed to prove increases in these cells, whose frequency even decreases in the course of the treatment (20, 35, 53, 79). This has prompted the hypothesis that OIT acts primarily through anergy, which designates T cell unresponsiveness to the antigen, or deletion, which denotes apoptosis of antigen-specific T cells, rather than a specific counterregulatory Treg response (20, 35, 36, 79). Additionally, while the clinical response to OIT is allergen-specific, this specificity of action may, at least in part, stem from the release of IL-2 by specific Th2 cells (27). This raises the question of whether immune suppression is more effective when regulatory and effector cells share the same antigen specificity and whether such specificity could enable more potent and localized control of inflammation (71). Indeed, a superior suppressive role of specific Treg cells could explain why continuous allergen exposure is required to maintain tolerance (27).

**Table 2:** Evolution of antigen-specific T cell responses in the course of immunotherapy (IT).

Methodology for detection	Clinical trial	Clinical outcome	Relevance to allergic disease	Reference
Peptide (Ara h 2) MHCII hexamer staining	Peanut OIT	Scalation to a target dose of 4000 mg peanut protein in 24 months, followed by 3 months of peanut avoidance	Reduction of antigen-specific Th2 and Treg cells and induction of anergic memory (CD28^-^K_i_-67^-^CD69^lo^) and non-allergic (IFNγ^+^) antigen specific CD4^+^ T cells only in the immune tolerant individuals (n= 5) comparted to pre-treatment and healthy controls (n= 7)	Ryan et al. (79)
Intracellular detection of CD154 after 6 h of stimulation	Randomized placebo controlled peanut OIT (CODIT)	Scalation to a target maintenance dose of 300 mg peanut protein in 20 weeks	Decreased Th2A frequency correlates with desensitization during peanut-OIT in the active group (n= 4) compared to placebo (n= 3)	Wambre et al. (34)
Surface expression of CD154 after 6 h of stimulation	Randomized placebo controlled peanut cutaneous IT (CoFAR6)	Viaskin peanut patch of 100 or 250 µg for 52 weeks followed by 250 µg in 130 weeks	Significant reduction in the frequency of peanut-responsive CD154^+^IL4^+^ cells or CD154^+^IL13^+^ compared to baseline (n= 74)	Berin et al. (38)
Surface expression of CD154 after 6 h of stimulation	Randomized egg OIT or backed egg (CoFAR7)	Scalation to a target maintenance dose of 2000 mg of egg white protein in 114 weeks	Baseline type 2 responses are significantly associated with oral immunotherapy failure (n= 92)	Berin et al. (38)
Surface expression of CD154 and CD137 after 20 h of stimulation	Randomized placebo controlled peanut OIT	Scalation to a target maintenance dose of 4000 mg of peanut protein in 44 weeks, a 12-week maintenance phase, and a 12-week avoidance phase	OIT decreases the frequency of CD154^+^ and CD137^+^ cells in the active group (n=9) compared to placebo (n= 3) OIT suppresses Th1 conv and Th2A-like clonotypes The baseline expression of Th2 signatures is not predictive, but the gene signatures present in Th1 and Th17 cells are, pointing at inflammation or altered gastrointestinal permeability	Monian et al. (20)
Surface expression of CD154 after 14 h of stimulation	Randomized placebo controlled peanut OIT (PALISADE)	Scalation to a target maintenance dose of 300 mg of peanut protein in 22 weeks and 6 months of maintenance	OIT decreases the frequency of pathogenic CRTH2^+^ peanut-specific cells in the absence of changes in the frequency of the CCR6^+^CD27^+^ subset (see **Table 1**) in the active group (n= 30) compared to placebo (n= 12) CRTH2^+^ peanut-specific cells at baseline correlate with specific IgG4 No differences in circulating peanut-specific Treg cells	Bajzik et al. (35)
Surface expression of CD154 and CD137 after 16 h of stimulation	Randomized placebo controlled peanut OIT (IMPACT)	Scalation to a target maintenance dose of 2000 mg of peanut protein in 30 weeks, a 104-week maintenance phase, and a 26-week avoidance phase	OIT decreases the frequency of pathogenic CRTH2^+^ peanut-specific cells, while CCR6^+^ cells showed a reciprocal relative increase in the active group (n= 21) compared to placebo (n= 8) Participants who experienced desensitization and remission (n= 8) had less than 20% CRTH2^+^ cells in total peanut-specific T cells at baseline Effector peanut-specific T cells at baseline correlate with specific IgE No variations in peanut-specific Treg cell frequency	Calise et al. (53)
Surface expression of CD154 and CD137 after 18 h of stimulation	Randomized placebo controlled intradermal IT with 7 Ara h 1 and Ara h 2 peptides	6 doses (150 nmol) of peanut peptides in 16 weeks, with determinations at week 21 and month 18	In the active group (n= 10) there was a decrease in the percentage of CD154^+^ST2^+^ Th2A cells and an increase in the percentage of CD154^+^CCR6^+^ Th17 cells expressing regulatory genes and regulatory markers within the peanut reactive CD4^+^ T cell population compared to placebo (n= 5) No changes in specific IgE or IgG4 levels or in skin prick tests Oral food challenges were not performed	Voskamp et al. (80)

IT, immunotherapy; OIT, oral immunotherapy.

## 6 Future prospects

More studies with larger cohorts are needed to outline the mechanisms underlying the interplay between specific pathogenic Th2 and Treg cells in food allergy and natural tolerance, as well as following OIT-induced desensitization in order to provide long-lasting benefits. It is envisaged that advances in protocols for T cell activation and antigen-MHC class II multimer preparation will make it possible to detect, with higher specificity, pathogenic and regulatory allergen-specific cells. In the meanwhile, the rapid development of technologies for analyzing immune responses with single-cell resolution that offer multiplexed characterization will expand functional and phenotypic analysis of these rare cells, even providing dynamic data or information on intercellular communication. Finally, access to the affected tissues in the gastrointestinal tract will help us understand more about allergic pathways and their regulatory factors.

## References

[1] Berin MC. Targeting type 2 immunity and the future of food allergy treatment. J Exp Med. (2023) 220:e20221104. doi: 10.1084/jem.20221104 36880703 PMC9997511

[2] Vandamme C Rytkönen-Nissinen M Lönnberg T Randell J Harvima RJ Kinnunen T. Single-cell characterization of dog allergen-specific T cells reveals TH2 heterogeneity in allergic individuals. J Allergy Clin Immunol. (2022) 149:1732–1743.e15. doi: 10.1016/j.jaci.2021.11.018 34863852

[3] Bacher P Schink C Teutschbein J Kniemeyer O Assenmacher M Brakhage AA. Antigen-reactive T cell enrichment for direct, high-resolution analysis of the human naive and memory Th cell repertoire. J Immunol. (2013) 190:3967–76. doi: 10.4049/jimmunol.1202221 23479226

[4] Prussin C Lee J Foster B. Eosinophilic gastrointestinal disease and peanut allergy are alternatively associated with IL-5^+^ and IL-5^-^ Th2 responses. J Allergy Clin Immunol. (2009) 124:1326–32.e6. doi: 10.1016/j.jaci.2009.09.048 20004787 PMC2994258

[5] Pascal M Konstantinou GN Masilamani M Lieberman J Sampson HA. In silico prediction of Ara h 2 T cell epitopes in peanut-allergic children. Clin Exp Allergy. (2013) 43:116–27. doi: 10.1111/cea.12014 23278886

[6] Prickett SR Voskamp AL Phan T Dacumos-Hill A Mannering SI Rolland JM. Ara h 1 CD4^+^ T cell epitope-based peptides: candidates for a peanut allergy therapeutic. Clin Exp Allergy. (2013) 43:684–97. doi: 10.1111/cea.12113 PMC3709139 23711131

[7] Syed A Garcia MA Lyu SC Bucayu R Kohli A Ishida S. Peanut oral immunotherapy results in increased antigen-induced regulatory T-cell function and hypomethylation of forkhead box protein 3 (FOXP3). J Allergy Clin Immunol. (2014) 133:500–10. doi: 10.1016/j.jaci.2013.12.1037 PMC4121175 24636474

[8] Wisniewski JA Commins SP Agrawal R Hulse KE Yu MD Cronin J. Analysis of cytokine production by peanut-reactive T cells identifies residual Th2 effectors in highly allergic children who received peanut oral immunotherapy. Clin Exp Allergy. (2015) 45:1201–13. doi: 10.1111/cea.12537 PMC4472497 25823600

[9] Kosoy R Agashe C Grishin A Leung DY Wood RA Sicherer SH. Transcriptional profiling of egg allergy and relationship to disease phenotype. PloS One. (2016) 11:e0163831. doi: 10.1371/journal.pone.0163831 27788149 PMC5082817

[10] Bacher P Scheffold A. The effect of regulatory T cells on tolerance to airborne allergens and allergen immunotherapy. J Allergy Clin Immunol. (2018) 142:1697–709. doi: 10.1016/j.jaci.2018.10.016 30527063

[11] Porebski G Piotrowicz-Wojcik K Spiewak R. ELISpot assay as a diagnostic tool in drug hypersensitivity reactions. J Immunol Methods. (2021) 495:113062. doi: 10.1016/j.jim.2021.113062 33940020

[12] Rust BJ Wambre E. Human immune monitoring techniques during food allergen immunotherapy. Curr Allergy Asthma Rep. (2017) 17:22. doi: 10.1007/s11882-017-0689-y 28361386

[13] Van Overtvelt L Wambre E Maillère B von Hofe E Louise A Balazuc AM. Assessment of Bet v 1-specific CD4^+^ T cell responses in allergic and nonallergic individuals using MHC class II peptide tetramers. J Immunol. (2008) 180:4514–22. doi: 10.4049/jimmunol.180.7.4514 18354173

[14] DeLong JH Simpson KH Wambre E James EA Robinson D Kwok WW. Ara h 1-reactive T cells in individuals with peanut allergy. J Allergy Clin Immunol. (2011) 127:1211–1218.e3. doi: 10.1016/j.jaci.2011.02.028 21459424 PMC3130623

[15] Birrueta G Tripple V Pham J Manohar M James EA Kwok WW. Peanut-specific T cell responses in patients with different clinical reactivity. PloS One. (2018) 13:e0204620. doi: 10.1371/journal.pone.0204620 30304054 PMC6179248

[16] Calise J Garabatos N Bajzik V Farrington M Robinson D Jeong D. Optimal human pathogenic TH2 cell effector function requires local epithelial cytokine signaling. J Allergy Clin Immunol. (2021) 148:867–875.e4. doi: 10.1016/j.jaci.2021.02.019 33662368 PMC8408285

[17] Bonvalet M Wambre E Moussu H Horiot S Kwok WW Louise A. Comparison between major histocompatibility complex class II tetramer staining and surface expression of activation markers for the detection of allergen-specific CD4^+^ T cells. Clin Exp Allergy. (2011) 41:821–9. doi: 10.1111/j.1365-2222.2011.03708.x 21418343

[18] Renand A Farrington M Whalen E Wambre E Bajzik V Chinthrajah S. Heterogeneity of Ara h component-specific CD4 T cell responses in peanut-allergic subjects. Front Immunol. (2018) 9:1408. doi: 10.3389/fimmu.2018.01408 29988522 PMC6026622

[19] Poloni C Schonhofer C Ivison S Levings MK Steiner TS Cook L. T-cell activation-induced marker assays in health and disease. Immunol Cell Biol. (2023) 101:491–503. doi: 10.1111/imcb.12636 36825901 PMC10952637

[20] Monian B Tu AA Ruiter B Morgan DM Petrossian PM Smith NP. Peanut oral immunotherapy differentially suppresses clonally distinct subsets of T helper cells. J Clin Invest. (2022) 132:e150634. doi: 10.1172/JCI150634 34813505 PMC8759778

[21] Chattopadhyay PK Gierahn TM Roederer M Love JC. Single-cell technologies for monitoring immune systems. Nat Immunol. (2014) 15:128–35. doi: 10.1038/ni.2796 PMC4040085 24448570

[22] Meier S Stark R Frentsch M Thiel A. The influence of different stimulation conditions on the assessment of antigen-induced CD154 expression on CD4^+^ T cells. Cytometry A. (2008) 73:1035–42. doi: 10.1002/cyto.a.20640 18785645

[23] Hegazy AN West NR Stubbington MJT Wendt E Suijker KIM Datsi A. Circulating and tissue-resident CD4^+^ T cells with reactivity to intestinal microbiota are abundant in healthy individuals and function is altered during inflammation. Gastroenterology. (2017) 153:1320–1337.e16. doi: 10.1053/j.gastro.2017.07.047 28782508 PMC5687320

[24] Frentsch M Arbach O Kirchhoff D Moewes B Worm M Rothe M. Direct access to CD4^+^ T cells specific for defined antigens according to CD154 expression. Nat Med. (2005) 11:1118–24. doi: 10.1038/nm1292 16186818

[25] Chattopadhyay PK Yu J Roederer M. Live-cell assay to detect antigen-specific CD4^+^ T-cell responses by CD154 expression. Nat Protoc. (2006) 1:1–6. doi: 10.1038/nprot.2006.1 17406204

[26] Reiss S Baxter AE Cirelli KM Dan JM Morou A Daigneault A. Comparative analysis of activation induced marker (AIM) assays for sensitive identification of antigen-specific CD4 T cells. PloS One. (2017) 12:e0186998. doi: 10.1371/journal.pone.0186998 29065175 PMC5655442

[27] Lozano-Ojalvo D Tyler SR Aranda CJ Wang J Sicherer S Sampson HA. Allergen recognition by specific effector Th2 cells enables IL-2-dependent activation of regulatory T-cell responses in humans. Allergy. (2023) 78:697–713. doi: 10.1111/all.15512 36089900 PMC10111618

[28] Yu W Zhou X Dunham D Lyu SC Manohar M Zhang W. Allergen-specific CD8^+^ T cells in peanut-allergic individuals. J Allergy Clin Immunol. (2019) 143:1948–52. doi: 10.1016/j.jaci.2019.01.011 PMC6510483 30682458

[29] Schoenbrunn A Frentsch M Kohler S Keye J Dooms H Moewes B. A converse 4-1BB and CD40 ligand expression pattern delineates activated regulatory T cells (Treg) and conventional T cells enabling direct isolation of alloantigen-reactive natural Foxp3^+^ Treg. J Immunol. (2012) 189:5985–94. doi: 10.4049/jimmunol.1201090 23162126

[30] Bacher P Heinrich F Stervbo U Nienen M Vahldieck M Iwert C. Regulatory T cell specificity directs tolerance versus allergy against aeroantigens in humans. Cell. (2016) 167:1067–1078.e16. doi: 10.1016/j.cell.2016.09.050 27773482

[31] Nowak A Lock D Bacher P Hohnstein T Vogt K Gottfreund J. CD137^+^CD154^-^ expression as a regulatory T cell (Treg)-specific activation signature for identification and sorting of stable human tregs from *in vitro* expansion cultures. Front Immunol. (2018) 9:199. doi: 10.3389/fimmu.2018.00199 29467769 PMC5808295

[32] Lemieux A Sannier G Nicolas A Nayrac M Delgado GG Cloutier R. Enhanced detection of antigen-specific T cells by a multiplexed AIM assay. Cell Rep Methods. (2024) 4:100690. doi: 10.1016/j.crmeth.2023.100690 38228152 PMC10831934

[33] Archila LD Jeong D Pascal M Bartra J Juan M Robinson D. Jug r 2-reactive CD4^+^ T cells have a dominant immune role in walnut allergy. J Allergy Clin Immunol. (2015) 136:983–992.e7. doi: 10.1016/j.jaci.2015.01.029 25772597 PMC4568181

[34] Wambre E Bajzik V DeLong JH O'Brien K Nguyen QA Speake C. A phenotypically and functionally distinct human Th2 cell subpopulation is associated with allergic disorders. Sci Transl Med. (2017) 9:eaam9171. doi: 10.1126/scitranslmed.aam9171 28768806 PMC5987220

[35] Bajzik V DeBerg HA Garabatos N Rust BJ Obrien KK Nguyen QA. Oral desensitization therapy for peanut allergy induces dynamic changes in peanut-specific immune responses. Allergy. (2022) 77:2534–48. doi: 10.1111/all.15276 PMC9356972 35266148

[36] Chiang D Chen X Jones SM Wood RA Sicherer SH Burks AW. Single-cell profiling of peanut-responsive T cells in patients with peanut allergy reveals heterogeneous effector Th2 subsets. J Allergy Clin Immunol. (2018) 141:2107–20. doi: 10.1016/j.jaci.2017.11.060 PMC5994177 29408715

[37] Berin MC Grishin A Masilamani M Leung DYM Sicherer SH Jones SM. Egg-specific IgE and basophil activation but not egg-specific T-cell counts correlate with phenotypes of clinical egg allergy. J Allergy Clin Immunol. (2018) 142:149–158.e8. doi: 10.1016/j.jaci.2018.01.044 29518422 PMC6282170

[38] Berin MC Agashe C Burks AW Chiang D Davidson WF Dawson P. Allergen-specific T cells and clinical features of food allergy: Lessons from CoFAR immunotherapy cohorts. J Allergy Clin Immunol. (2022) 149:1373–1382.e12. doi: 10.1016/j.jaci.2021.09.029 34653515 PMC8995337

[39] Gowthaman U Chen JS Zhang B Flynn WF Lu Y Song W. Identification of a T follicular helper cell subset that drives anaphylactic IgE. Science. (2019) 365:eaaw6433. doi: 10.1126/science.aaw6433 31371561 PMC6901029

[40] Weissler KA Rasooly M DiMaggio T Bolan H Cantave D Martino D. Identification and analysis of peanut-specific effector T and regulatory T cells in children allergic and tolerant to peanut. J Allergy Clin Immunol. (2018) 141:1699–1710.e7. doi: 10.1016/j.jaci.2018.01.035 29454004 PMC5938104

[41] Lai CL Campbell DE Palmer DJ Makrides M Santner-Nanan B Gold M. Longitudinal egg-specific regulatory T- and B-cell development: Insights from primary prevention clinical trials examining the timing of egg introduction. Allergy. (2021) 76:1385–97. doi: 10.1111/all.14621 33040362

[42] Lewis SA Sutherland A Soldevila F Westernberg L Aoki M Frazier A. Identification of cow milk epitopes to characterize and quantify disease-specific T cells in allergic children. J Allergy Clin Immunol. (2023) 152:1196–209. doi: 10.1016/j.jaci.2023.07.020 PMC10846667 37604312

[43] Renand A Newbrough S Wambre E DeLong JH Robinson D Kwok WW. Arginine kinase Pen m 2 as an important shrimp allergen recognized by TH2 cells. J Allergy Clin Immunol. (2014) 134:1456–1459.e7. doi: 10.1016/j.jaci.2014.07.048 25224098 PMC4349443

[44] Archila LD Chow IT McGinty JW Renand A Jeong D Robinson D. Ana o 1 and Ana o 2 cashew allergens share cross-reactive CD4^+^ T cell epitopes with other tree nuts. Clin Exp Allergy. (2016) 46:871–83. doi: 10.1111/cea.12746 PMC5502816 27129138

[45] Ruiter B Smith NP Monian B Tu AA Fleming E Virkud YV. Expansion of the CD4^+^ effector T-cell repertoire characterizes peanut-allergic patients with heightened clinical sensitivity. J Allergy Clin Immunol. (2020) 145:270–82. doi: 10.1016/j.jaci.2019.09.033 PMC6949413 31654649

[46] Blom LH Juel-Berg N Larsen LF Hansen KS Poulsen LK. Circulating allergen-specific Th2 lymphocytes: CCR4^+^ rather than CLA^+^ is the predominant phenotype in peanut-allergic subjects. J Allergy Clin Immunol. (2018) 141:1498–1501.e5. doi: 10.1016/j.jaci.2017.10.037 29225086

[47] Kurioka A Cosgrove C Simoni Y van Wilgenburg B Geremia A Björkander S. CD161 defines a functionally distinct subset of pro-inflammatory natural killer cells. Front Immunol. (2018) 9:486. doi: 10.3389/fimmu.2018.00486 29686665 PMC5900032

[48] Kratchmarov R Djeddi S Dunlap G He W Jia X Burk CM. TCF1-LEF1 co-expression identifies a multipotent progenitor cell (TH2-MPP) across human allergic diseases. Nat Immunol. (2024) 25:902–15. doi: 10.1038/s41590-024-01803-2 PMC11849131 38589618

[49] Lozano-Ojalvo D Chen X Kazmi W Menchén-Martínez D Pérez-Rodríguez L Fernandes-Braga W. Differential T follicular helper cell phenotypes distinguish IgE-mediated milk allergy from eosinophilic esophagitis in children. J Allergy Clin Immunol. (2025) 155:909-22. doi: 10.1016/j.jaci.2024.09.024 PMC11895100 39389123

[50] Brough HA Cousins DJ Munteanu A Wong YF Sudra A Makinson K. IL-9 is a key component of memory Th cell peanut-specific responses from children with peanut allergy. J Allergy Clin Immunol. (2014) 134:1329–1338.e10. doi: 10.1016/j.jaci.2014.06.032 25112699

[51] Pettipher R. The roles of the prostaglandin D_2_ receptors DP_1_ and CRTH2 in promoting allergic responses. Br J Pharmacol. (2008) 153:S191–199. doi: 10.1038/sj.bjp.0707488 PMC2268053 17965752

[52] Mitson-Salazar A Yin Y Wansley DL Young M Bolan H Arceo S. Hematopoietic prostaglandin D synthase defines a proeosinophilic pathogenic effector human Th2 cell subpopulation with enhanced function. J Allergy Clin Immunol. (2016) 137:907–918.e9. doi: 10.1016/j.jaci.2015.08.007 26431580

[53] Calise J DeBerg H Garabatos N Khosa S Bajzik V Calderon LB. Distinct trajectories distinguish antigen-specific T cells in peanut-allergic individuals undergoing oral immunotherapy. J Allergy Clin Immunol. (2023) 152:155–166.e9. doi: 10.1016/j.jaci.2023.03.020 37003475 PMC10330178

[54] Oboki K Ohno T Saito H Nakae S. Th17 and allergy. Allergol Int. (2008) 57:121–34. doi: 10.2332/allergolint.R-07-160 18427165

[55] Tortola L Jacobs A Pohlmeier L Obermair FJ Ampenberger F Bodenmiller B. High-dimensional T helper cell profiling reveals a broad diversity of stably committed effector states and uncovers interlineage relationships. Immunity. (2020) 53:597–613.e6. doi: 10.1016/j.immuni.2020.07.001 32735846

[56] Noval Rivas M Burton OT Wise P Charbonnier LM Georgiev P Oettgen HC. Regulatory T cell reprogramming toward a Th2-cell-like lineage impairs oral tolerance and promotes food allergy. Immunity. (2015) 42:512–23. doi: 10.1016/j.immuni.2015.02.004 PMC4366316 25769611

[57] Prince BT Devonshire AL Erickson KA Bergerson J Fuleihan D Szychlinski C. Regulatory T-cell populations in children are affected by age and food allergy diagnosis. J Allergy Clin Immunol. (2017) 140:1194–1196.e16. doi: 10.1016/j.jaci.2017.04.039 28549988 PMC5777506

[58] Perezabad L López-Abente J Alonso-Lebrero E Seoane E Pion M Correa-Rocha R. The establishment of cow's milk protein allergy in infants is related with a deficit of regulatory T cells (Treg) and vitamin D. Pediatr Res. (2017) 81:722–30. doi: 10.1038/pr.2017.12 28099424

[59] Qamar N Fishbein AB Erickson KA Cai M Szychlinski C Bryce PJ. Naturally occurring tolerance acquisition to foods in previously allergic children is characterized by antigen specificity and associated with increased subsets of regulatory T cells. Clin Exp Allergy. (2015) 45:1663–72. doi: 10.1111/cea.12570 PMC4607624 25989379

[60] Karlsson MR Rugtveit J Brandtzaeg P. Allergen-responsive CD4+CD25+ regulatory T cells in children who have outgrown cow's milk allergy. J Exp Med. (2004) 199:1679–88. doi: 10.1084/jem.20032121 PMC2212808 15197226

[61] Shreffler WG Wanich N Moloney M Nowak-Wegrzyn A Sampson HA. Association of allergen-specific regulatory T cells with the onset of clinical tolerance to milk protein. J Allergy Clin Immunol. (2009) 123:43–52.e7. doi: 10.1016/j.jaci.2008.09.051 19130927

[62] Seumois G Ramírez-Suástegui C Schmiedel BJ Liang S Peters B Sette A. Single-cell transcriptomic analysis of allergen-specific T cells in allergy and asthma. Sci Immunol. (2020) 5:eaba6087. doi: 10.1126/sciimmunol.aba6087 32532832 PMC7372639

[63] Stoumpos A Heine G Saggau C Scheffold A. The role of allergen-specific regulatory T cells in the control of allergic disease. Curr Opin Immunol. (2025) 92:102509. doi: 10.1016/j.coi.2024.102509 39642798

[64] Pellerin L Jenks JA Chinthrajah S Dominguez T Block W Zhou X. Peanut-specific type 1 regulatory T cells induced *in vitro* from allergic subjects are functionally impaired. J Allergy Clin Immunol. (2018) 141:202–213.e8. doi: 10.1016/j.jaci.2017.05.045 28689791

[65] Ding Y Xu J Bromberg JS. Regulatory T cell migration during an immune response. Trends Immunol. (2012) 33:174–80. doi: 10.1016/j.it.2012.01.002 PMC3319498 22305714

[66] Liu Z Gerner MY Van Panhuys N Levine AG Rudensky AY Germain RN. Immune homeostasis enforced by co-localized effector and regulatory T cells. Nature. (2015) 528:225–30. doi: 10.1038/nature16169 PMC4702500 26605524

[67] Wen T Aronow BJ Rochman Y Rochman M Kc K Dexheimer PJ. Single-cell RNA sequencing identifies inflammatory tissue T cells in eosinophilic esophagitis. J Clin Invest. (2019) 129:2014–28. doi: 10.1172/JCI125917 PMC6486341 30958799

[68] Noval Rivas M Burton OT Oettgen HC Chatila T. IL-4 production by group 2 innate lymphoid cells promotes food allergy by blocking regulatory T-cell function. J Allergy Clin Immunol. (2016) 138:801–811.e9. doi: 10.1016/j.jaci.2016.02.030 27177780 PMC5014699

[69] Dikiy S Ghelani AP Levine AG Martis S Giovanelli P Wang ZM. Terminal differentiation and persistence of effector regulatory T cells essential for preventing intestinal inflammation. Nat Immunol. (2025) 26:444-58. doi: 10.1038/s41590-024-02075-6 PMC11876075 39905200

[70] Okeke EB Uzonna JE. The pivotal role of regulatory T cells in the regulation of innate immune cells. Front Immunol. (2019) 10:680. doi: 10.3389/fimmu.2019.00680 31024539 PMC6465517

[71] Selck C Dominguez-Villar M. Antigen-specific regulatory T cell therapy in autoimmune diseases and transplantation. Front Immunol. (2021) 12:661875. doi: 10.3389/fimmu.2021.661875 34054826 PMC8160309

[72] Mondoulet L Dioszeghy V Busato F Plaquet C Dhelft V Bethune K. Gata3 hypermethylation and Foxp3 hypomethylation are associated with sustained protection and bystander effect following epicutaneous immunotherapy in peanut-sensitized mice. Allergy. (2019) 74:152–64. doi: 10.1111/all.13479 PMC6585762 29779209

[73] Moldaver DM Bharhani MS Wattie JN Ellis R Neighbour H Lloyd CM. Amelioration of ovalbumin-induced allergic airway disease following Der p 1 peptide immunotherapy is not associated with induction of IL-35. Mucosal Immunol. (2014) 7:379–90. doi: 10.1038/mi.2013.56 23945544

[74] Hong SW Krueger PD Osum KC Dileepan T Herman A Mueller DL. Immune tolerance of food is mediated by layers of CD4^+^ T cell dysfunction. Nature. (2022) 607:762–8. doi: 10.1038/s41586-022-04916-6 PMC10336534 35794484

[75] Blum JE Kong R Schulman EA Chen FM Upadhyay R Romero-Meza G. Discovery and characterization of dietary antigens in oral tolerance. bioRxiv. (2024), 2024.05.26.593976. doi: 10.1101/2024.05.26.593976 PMC13242657 41790933

[76] Chauhan SK Bartolomé Casado R Landsverk OJB Johannessen H Phung D Nilsen HR. Human small intestine contains 2 functionally distinct regulatory T-cell subsets. J Allergy Clin Immunol. (2023) 152:278–289.e6. doi: 10.1016/j.jaci.2023.02.030 36893861

[77] Palomares O Rückert B Jartti T Kücüksezer UC Puhakka T Gomez E. Induction and maintenance of allergen-specific FOXP3^+^ Treg cells in human tonsils as potential first-line organs of oral tolerance. J Allergy Clin Immunol. (2012) 129:510–520, 520. doi: 10.1016/j.jaci.2011.09.031 22051696

[78] Hanif T Ivaska LE Ahmad F Tan G Mikola E Puhakka T. Tonsillar transcriptional profiles in atopic and non-atopic subjects. Allergy. (2023) 78:522–36. doi: 10.1111/all.15458 PMC10087516 35899482

[79] Ryan JF Hovde R Glanville J Lyu SC Ji X Gupta S. Successful immunotherapy induces previously unidentified allergen-specific CD4^+^ T-cell subsets. Proc Natl Acad Sci U S A. (2016) 113:E1286–95. doi: 10.1073/pnas.1520180113 PMC4780622 26811452

[80] Voskamp AL Khosa S Phan T DeBerg HA Bingham J Hew M. Phase 1 trial supports safety and mechanism of action of peptide immunotherapy for peanut allergy. Allergy. (2024) 79:485–98. doi: 10.1111/all.15966 38112286

